# Identification of Genomic Loci Responsible for the Formation of Nuclear Domains Using Lampbrush Chromosomes

**DOI:** 10.3390/ncrna6010001

**Published:** 2019-12-25

**Authors:** Alla Krasikova, Tatiana Kulikova

**Affiliations:** Saint-Petersburg State University, 7/9 Universitetskaya emb., 199034 St. Petersburg, Russia

**Keywords:** nuclear domain, nuclear body, lampbrush chromosomes, microdissection, FISH-mapping, non-coding RNA, architectural RNA

## Abstract

In the cell nuclei, various types of nuclear domains assemble as a result of transcriptional activity at specific chromosomal loci. Giant transcriptionally active lampbrush chromosomes, which form in oocyte nuclei of amphibians and birds enable the mapping of genomic sequences with high resolution and the visualization of individual transcription units. This makes avian and amphibian oocyte nuclei an advantageous model for studying locus-specific nuclear domains. We developed two strategies for identification and comprehensive analysis of the genomic loci involved in nuclear domain formation on lampbrush chromosomes. The first approach was based on the sequential FISH-mapping of BAC clones containing genomic DNA fragments with a known chromosomal position close to the locus of a nuclear domain. The second approach involved mechanical microdissection of the chromosomal region adjacent to the nuclear domain followed by the generation of FISH-probes and DNA sequencing. Furthermore, deciphering the DNA sequences from the dissected material by high throughput sequencing technologies and their mapping to the reference genome helps to identify the genomic region responsible for the formation of the nuclear domain. For those nuclear domains structured by nascent transcripts, identification of genomic loci of their formation is a crucial step in the identification of scaffold RNAs.

## 1. Introduction

According to modern knowledge on the mechanisms which underlie the formation of nuclear bodies, the assembly of certain nuclear domains is nucleated by RNA at the transcription loci [1,2]. Furthermore, non-coding RNA may serve as a scaffold for gathering specific ribonucleoprotein (RNP) complexes, which helps to increase their concentration to a level sufficient for liquid-liquid phase separation [3,4]. Recent studies reveal that RNA has a major role in the regulation of phase separation of RNP-complexes. Repetitive motifs in RNA sequences are involved in RNA-driven and protein-independent RNP coalescence resulting in the formation of nuclear foci [5]. On the other hand, maintenance of high RNA concentration prevents the formation of insoluble RNP complexes [6]. Studies of the nucleolus reassembly after stress suggest that RNA species, which initiates nuclear domain formation also drives transfer of proteins from disassembling transient nuclear bodies to its parental reassembling nuclear domain [7].

A nucleolus forming around transcribing clusters of ribosomal RNA genes is a vivid example of a ubiquitous locus-specific nuclear domain. However, recent studies prove that the maintenance of nucleolus integrity requires non-ribosomal RNA polymerase II transcripts [8]. Another well studied nuclear domain dependent on architectural RNA (arcRNA)—the paraspeckle—assembles at the locus of NEAT1_2/Menε/β long non-coding RNA transcription [9]. The number of arcRNA species for well-known or newly characterized nuclear domains grows steadily [10,11,12,13,14,15,16].

Coilin-containing nuclear bodies represent another type of nuclear domain with locus-specific localization. Coilin-containing nuclear bodies form in close association with the transcription units of RNA species requiring specific processing machinery (snRNA genes, snoRNA genes and histone gene clusters), although they are not structured by the nascent transcripts themselves [17].

## 2. Locus-Specific Nuclear Domains on Lampbrush Chromosomes

**Figure 1:**
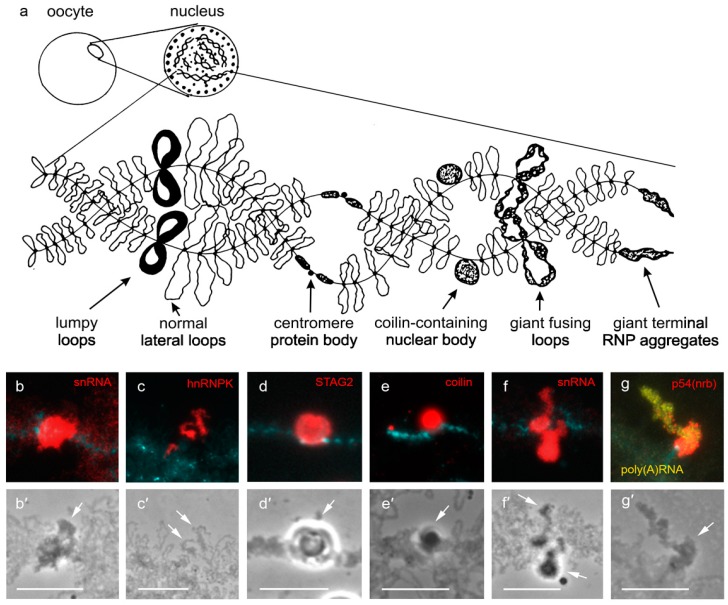
Locus-specific nuclear domains on lampbrush chromosomes. (**a**) Schematic drawing of a lampbrush chromosome with various locus-specific nuclear bodies and domains. Lumpy loops, ‘normal’ lateral loops, centromere protein bodies, coilin-containing nuclear bodies, giant fusing loops and giant terminal RNP aggregates (GITERA) are shown. (**b**) Lumpy loops forming on lampbrush chromosome K of the lake frog (*Pelophylax ridibundus*) after immunostaining with antibodies against splicing snRNA (red). (**c**) ‘Normal’ lateral loops bearing transcription units of 41 bp tandem repeat CNM on lampbrush chromosome 3 of chicken (*Gallus gallus domesticus*) after immunostaining with antibodies against hnRNP K (red). (**d**) Centromere protein body on lampbrush chromosome of the chaffinch (*Fringilla coelebs*) after immunostaining with antibodies against STAG2 protein of cohesin complex (red). (**e**) Coilin-containing body on lampbrush chromosome B of the lake frog (*P. ridibundus*) revealed by immunostaining with antibodies against coilin (red). (**f**) Giant fusing loops forming on lampbrush chromosome H of the lake frog (*P. ridibundus*) after immunostaining with antibodies against splicing snRNA (red). (**g**) GITERA on lampbrush micro-chromosome of pigeon (*Columba livia*) accumulate poly (A) + RNA revealed by FISH with oligo-dT (yellow) and paraspeckle protein p54 (nrb)/NONO (red). Chromosomes are counterstained with DAPI (cyan). (**b′**–**g′**)—corresponding phase contrast images. Scale bars—10 μm.

The giant lampbrush chromosomes represent transcriptionally active meiotic half-bivalents forming at the diplotene stage of meiosis in oocytes of non-mammalian species; lampbrush chromosomes are the most well-studied in amphibians and birds [18,19]. Similar to mitotic metaphase chromosomes, lampbrush chromosomes can be isolated as spread on a slide. At the same time, lampbrush chromosomes have a distinct chromomere-loop organization and are a dozen times more extended than metaphase chromosomes (Figure 1a) [19,20]. Thus, using Fluorescence in situ hybridization (FISH) on lampbrush chromosomes makes it possible to map genomic sequences with high cytogenetic resolution and to supplement and/or to correct genome sequence assembly [21,22]. The high level of transcriptional activity on lateral loops of lampbrush chromosomes allows the visualization of nascent transcripts and components of RNA processing machinery at the level of individual transcription units [23,24]. Another benefit of the lampbrush stage of oogenesis is the larger sized nuclear bodies and domains which form on lampbrush chromosomes. For instance, the length of the ‘giant terminal RNP aggregates’ (GITERA) (Figure 1g) forming on avian lampbrush chromosomes varies from 10 to 100 μm depending on the species [25,26,27,28]. Coilin-containing nuclear bodies in the amphibian oocyte nucleus reach 5–10 μm in diameter [18,29] versus 0.5–1.8 μm in the interphase nucleus [30]. A high level of chromosome decondensation with the distinct chromomere-loop organization facilitates precise mechanical microdissection of locus-specific nuclear domains on lampbrush chromosomes [31]. As a whole, this makes lampbrush chromosomes a useful model for studying all known types of locus-specific nuclear domains and for discovering nuclear domains not yet described in interphase nuclei. Notably, the two types of coilin-containing spherical bodies—‘pearls’, associated with RNA polymerase III transcription units, and histone locus body—were first described in the studies of the oocyte nucleus and, specifically, the lampbrush chromosomes (Figure 1e) [29,32,33]. In addition to the coilin-containing nuclear bodies, there are a number of various nuclear domains forming on lampbrush chromosomes; and the majority of them supposedly have equivalents in the interphase nucleus (Figure 1a).

The centromere protein bodies represent spherical bodies attached to the centromere regions of lampbrush chromosomes and evidently belong to meiosis-specific nuclear domains. Centromere protein bodies lack components of the RNA-processing machinery and accumulate chromosome architectural proteins: DNA topoisomerase II [34] and cohesin complex proteins (Figure 1d) [35]. The accumulating evidence indicates that the formation of centromere protein bodies is RNA-independent: (1) the centromere protein bodies lack RNA; (2) the locus of centromere protein body formation (central domain of the centromeres) is not involved in transcription [36,37].

Another group of locus-specific domains on lampbrush chromosomes combines RNP-rich non-spherical structures forming in association with specific transcription units. Immunofluorescence and 3D RNA FISH on isolated oocyte nuclei revealed that certain lampbrush lateral loops or loci-specific RNP aggregates manifest as nuclear domains since they accumulate specific RNA-binding proteins and RNA species as opposed to the nucleoplasm [38]. For example, lumpy loops (Figure 1b) and GITERA (Figure 1g) forming at specific transcriptionally active loci of lampbrush chromosomes appear as SC35 domains in 3D [38]. At the same time, transcription units of 41 bp tandem repeats (CNM and PO41) forming lateral loops with ‘normal’ morphology [39] represent distinct nuclear domains enriched with heterogeneous nuclear ribonucleoprotein K (hnRNP K) (Figure 1c) [38]. Further studies of PO41 repeat transcription in chicken somatic tissues and during embryo development demonstrated that PO41 RNAs form ubiquitous nuclear domain during interphase [40,41]. We believe that non-spherical RNP-rich structures, which form at specific transcriptionally active loci of lampbrush chromosomes belong to nuclear domains structured by the RNA scaffold.

## 3. Approaches to Identification of Genomic Loci Responsible for Nuclear Domains Formation

Several genome-wide approaches for identification of genomic loci proximal to nuclear domains were recently developed for interphase nuclei. These approaches were used to study the 3D genomic environment of nuclear speckles [42,43,44], nucleolus [44] and Cajal bodies [45]. Alternatively, cytomolecular [46] and single-cell approaches [47,48] were developed for identification of genomic loci adjacent to the promyelocytic leukemia body and histone locus body. These studies suggest that nuclear domains behave as ‘hubs’ for genomic loci which they regulate. In case of Cajal bodies or histone locus bodies, identified nuclear body-interacting genomic loci coincided to the loci of their formation [45,47]. However, in other cases it seems challenging to identify a genomic locus defining nuclear domain formation among other genomic sequences located close to a nuclear domain in interphase nucleus.

**Figure 2:**
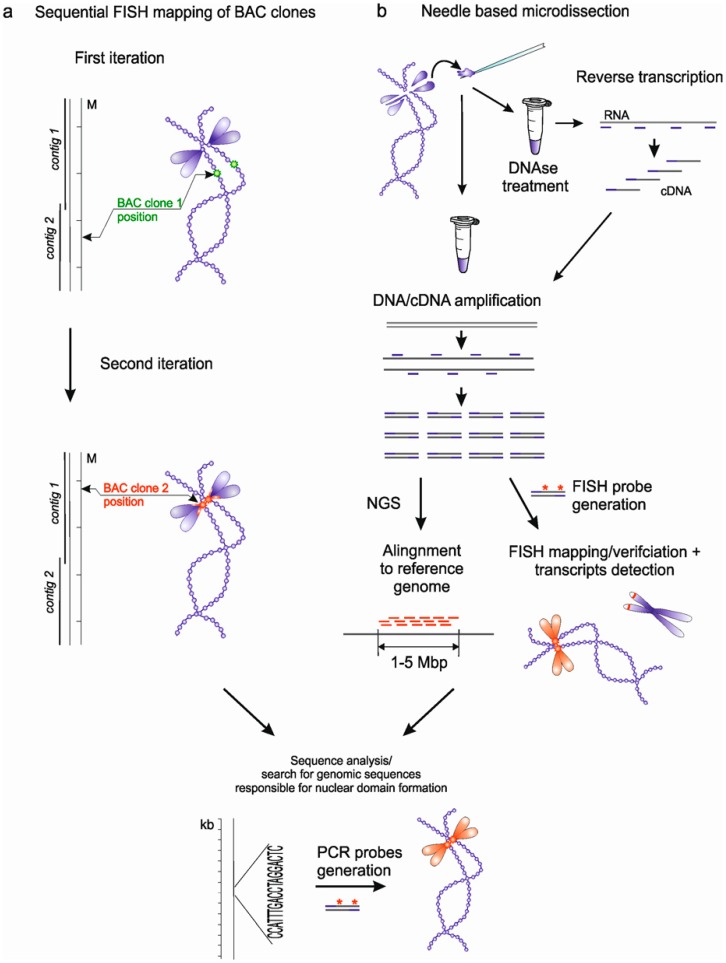
Scheme represents the two approaches developed for identification of the genomic loci and scaffold RNAs responsible for nuclear domain formation. (**a**) Sequential FISH-mapping of BAC clones containing genomic DNA fragments on lampbrush chromosomes to narrow down the genomic region of nuclear domain formation followed by bioinformatics analysis and scaffold RNAs identification. (**b**) Mechanical microdissection of chromomeres at the base of the nuclear domain on lampbrush chromosomes followed by generation of FISH-probes, high throughput sequencing and genome mapping of dissected DNA fragments.

Recently, we developed two strategies for analyzing the loci of nuclear domain formation on giant lampbrush chromosomes. The first approach was based on sequential FISH mapping of BAC clones containing genomic DNA fragments with a known position in the genome and located close to the site of the formation of a nuclear domain or marker structure in order to narrow the area of subsequent bioinformatics search and analysis of genomic loci (Figure 2a). This approach was, in particular, successfully applied to the analysis of the locus of lumpy loops formation on chicken lampbrush chromosome 2 (lumpy loops 2) [38,49]. Lumpy loops brightly fluoresce when stained with RNA-specific fluorescent dyes, representing a unit of the RNP-matrix enriched in RNA. Using sequential FISH-mapping of a number of BAC clones that contain fragments of chicken genomic DNA, we determined that lumpy loops 2 were formed as a result of transcription of the new bioinformatically identified tandem repeat LL2R [38,49]. Thus, using this approach, we demonstrated that transcription of non-coding RNA derived from tandem repeats could lead to the formation of nuclear domains enriched in certain RNP complexes.

The second suggested approach was based on mechanical microdissection of chromosome regions adjacent to a particular nuclear structure, followed by in vitro amplification of DNA from the dissected material, FISH-mapping and high throughput sequencing of the obtained DNA samples (Figure 2b). We demonstrated that lampbrush chromosome microdissection provides a significant advantage for a detailed study of nuclear structures or domains that are formed in association with particular genomic regions [31]. The developed approach allows one to obtain an extremely small amount of nucleic acids from the dissected material, suitable for subsequent PCR amplification. FISH mapping of DNA samples obtained by microdissection of individual lampbrush chromomeres (compacted chromatin domains) on metaphase chromosomes showed a surprisingly high specificity and brightness of the obtained fluorescence probes [31]. Thus, microdissection is a reliable technique that gives the opportunity to obtain DNA samples from individual marker structures formed at a specific lampbrush chromosome locus.

Subsequently, deciphering the DNA sequence from the dissected material using the next generation sequencing technologies and mapping of sequenced DNA fragments to the reference genome allows for the identification of genomic loci occupying 1.5–5 Mb [31,50]. One of the examples is the identification of genomic sequences at the basis of lumpy loops on chicken lampbrush chromosome 3 (lumpy loops 3). The microdissection approach allows not only for the analysis of the genomic locus of nuclear domain formation, but also the RNA composition of the domain itself. RNA from the dissected domain can be reverse transcribed with random hexamer primer to gain cDNA, which then can be amplified with degenerate primers and used for cDNA probe generation and sequencing (Figure 2b) [31].

It is important to emphasize that the use of such an integrated approach will accurately determine the genomic position of the nuclear domain formation, and then analyze its genomic and epigenetic characteristics. Moreover, great efforts should be given to the subsequent bioinformatics and functional analysis of the established genomic loci followed by identification of the transcripts involved in nuclear domain formation. At the moment, application of the aforementioned approaches is restricted to species with a lampbrush stage during oogenesis. However, in prospect, these approaches can be extended to mammalian species with artificial induction of lampbrush chromosomes formation using sperm head injection into the amphibian oocyte nucleus [51].
